# Supplementary material for: Substantial impact of post-vaccination contacts on cumulative infections during viral epidemics
Source: F1000Res. 2021 Aug 26;10:315. Originally published 2021 Apr 23. [Version 2] doi: 10.12688/f1000research.52341.2 (PMC8406440; doi:10.12688/f1000research.52341.2)
Supplement: Peer review history

# Peer review: Reviewer response for version 2

**DOI:** 10.5256/f1000research.76677.r92849

**Published:** 2021-08-26

**Authors:** Sebastian Maximilian Schloer

**Affiliations:** 1 Center for Molecular Biology of Inflammation, Institute of Medical Biochemistry, University of Muenster, Muenster, Germany

The authors have addressed all concerns adequately and improved the quality of the manuscript and its readability. I recommend the manuscript for indexing.

Is the work clearly and accurately presented and does it cite the current literature?

Yes

If applicable, is the statistical analysis and its interpretation appropriate?

Partly

Are all the source data underlying the results available to ensure full reproducibility?

Yes

Is the study design appropriate and is the work technically sound?

Yes

Are the conclusions drawn adequately supported by the results?

Yes

Are sufficient details of methods and analysis provided to allow replication by others?

Partly

Reviewer Expertise:

Virology, pharmacology, zoonotic diseases

I confirm that I have read this submission and believe that I have an appropriate level of expertise to confirm that it is of an acceptable scientific standard.

# Peer review: Reviewer response for version 2

**DOI:** 10.5256/f1000research.76677.r92848

**Published:** 2021-08-26

**Authors:** Fyodor A. Kondrashov

**Affiliations:** 1 Institute of Science and Technology, Vienna, Austria

I thank the authors for addressing my previous comments and have no further remarks.

Is the work clearly and accurately presented and does it cite the current literature?

Partly

If applicable, is the statistical analysis and its interpretation appropriate?

Yes

Are all the source data underlying the results available to ensure full reproducibility?

Yes

Is the study design appropriate and is the work technically sound?

Yes

Are the conclusions drawn adequately supported by the results?

Partly

Are sufficient details of methods and analysis provided to allow replication by others?

Yes

Reviewer Expertise:

Evolution

I confirm that I have read this submission and believe that I have an appropriate level of expertise to confirm that it is of an acceptable scientific standard.

# Peer review: Reviewer response for version 1

**DOI:** 10.5256/f1000research.55607.r83821

**Published:** 2021-08-12

**Authors:** Fyodor A. Kondrashov

**Affiliations:** 1 Institute of Science and Technology, Vienna, Austria

The ongoing SARS-CoV-2 pandemic is driving the need to explore a new set of SIR models. One new feature that has not been considered previously is the impact of the state of individuals in the model that have just been vaccinated (inoculated) but have not yet achieved full immunity.

 The authors took a classical SIR model and expanded it to include three new states: Infected with an Escape variant (E), Inoculated with the vaccine (I) and Inoculated but subsequently infected (M). The authors then explored the dynamics influenced by different parameters in this model. First, the higher contribution of Inoculated individuals to produce an Escaped variant. Second, a parameter that models limited interaction of inoculated hosts with others in the population. Third, the rate of vaccination.

 In light of the ongoing debates on infection rate of those individuals that have been vaccinated with currently available vaccines against SARS-CoV-2, this study appears particularly timely and with potential to provide critical insight to contribution of the Inoculated but not fully vaccinated to the ongoing dynamics. However, some aspects of this model potentially reduce its applicability. Nevertheless, as a theoretical, rather than an empirical, model it does present an interesting view of the dynamics based on the model’s structure and assumptions, and even suggests a specific chokepoint of Escape variant evolution that may be controlled with simple policy solutions (the contact behavior of the Inoculated).

 I have but two general criticisms, mostly related to presentation rather than design.

 First, the presentation of the model made it challenging to understand the setup. Figure 1A could be more useful (for example, it could introduce the states I, F, M, etc), and it could even represent the transition parameters. The naming of the states departs from the SIR model, (so I is not Infected anymore, but Inoculated), which is fine, but added to the confusion. Finally, I was not a big fan of the equation representations. The authors have chosen to represent the equations in the shortest possible form. I prefer representation where each part of the equation reflects some transition state. To checkout the current equations I had to expand all of them and make my own determination of each transition state and how it fits to Figure 1A. Perhaps a more mathematically savvy reader would not have had to do it.

 Second, as a theoretical model, it aims to describe some new understanding of interaction of parameters within the limitations of the model. A very successful example of this in the model is the analysis of the higher contribution of the Inoculated and Inoculated Infected states to dynamics and the introduction of another parameter, q, which limits the interaction of the Inoculated with other individuals. So, it explores the impact of the news states in the model and then introduces a specific way to mitigate their dangerous impact, which has important policy implications (granted that the underlying assumptions hold up). However, some aspects of the model cannot be applied directly to policy. Specifically, in several places of the manuscript the authors talk about the need for the determination of “optimal vaccination strategy”. While the model does explore vaccination rate as a parameter, I do not believe that their model is applicable to determination of a vaccine strategy at all. The reason for that is because a vaccine strategy considers some other parameters that are absent in the model. Specifically, an optimal vaccination strategy would consider, and probably prioritize, the short/medium term mortality rate and the hospital burden, something that is absent here.

 The design of the model in the confines of the classical SIR structure (where the pandemic geos through the population without any negative feedback loops leading to waves) also makes it unrealistic in providing an optimization of policy. In sum, the conceptual description of the model, and the subsequent discussion, should avoid references to quantitative optimization of potential policy decisions that are likely to be influenced by parameters and outcomes that are not inherent in the model.

**Minor issues:**

 Equation of V’ has a missing parenthesis.

Is the work clearly and accurately presented and does it cite the current literature?

Partly

If applicable, is the statistical analysis and its interpretation appropriate?

Yes

Are all the source data underlying the results available to ensure full reproducibility?

Yes

Is the study design appropriate and is the work technically sound?

Yes

Are the conclusions drawn adequately supported by the results?

Partly

Are sufficient details of methods and analysis provided to allow replication by others?

Yes

Reviewer Expertise:

Evolution

I confirm that I have read this submission and believe that I have an appropriate level of expertise to confirm that it is of an acceptable scientific standard, however I have significant reservations, as outlined above.

# Response

**Published:** 2021-08-16

**Authors:** Eugene Koonin

**Affiliations:** National Center for Biotechnology Information (NCBI), National Institutes of Health (NIH), USA

**We thank the reviewer for their interest and constructive comments. The model presented has 7 compartments. There are two compartments which represent uninfected, vaccinated hosts: those recently inoculated (I) and those who have “matured” into the fully vaccinated compartment.**

 In light of the ongoing debates on infection rate of those individuals that have been vaccinated with currently available vaccines against SARS-CoV-2, this study appears particularly timely and with potential to provide critical insight to contribution of the Inoculated but not fully vaccinated to the ongoing dynamics. However, some aspects of this model potentially reduce its applicability. Nevertheless, as a theoretical, rather than an empirical, model it does present an interesting view of the dynamics based on the model’s structure and assumptions, and even suggests a specific chokepoint of Escape variant evolution that may be controlled with simple policy solutions (the contact behavior of the Inoculated).

**We thank the reviewer again for their interest. This is precisely our motivation and we completely acknowledge the limitations of our model which is theoretical not empirical. As discussed below, we have modified the language in the text to emphasize this point.**

 I have but two general criticisms, mostly related to presentation rather than design.

 First, the presentation of the model made it challenging to understand the setup. Figure 1A could be more useful (for example, it could introduce the states I, F, M, etc), and it could even represent the transition parameters. The naming of the states departs from the SIR model, (so I is not Infected anymore, but Inoculated), which is fine, but added to the confusion. Finally, I was not a big fan of the equation representations. The authors have chosen to represent the equations in the shortest possible form. I prefer representation where each part of the equation reflects some transition state. To checkout the current equations I had to expand all of them and make my own determination of each transition state and how it fits to Figure 1A. Perhaps a more mathematically savvy reader would not have had to do it.

**We apologize for the confusion associated with the lack of detail in Figure 1A, which has been modified to include the state abbreviations (I,F,M, etc.) and the transition rates.**

 Second, as a theoretical model, it aims to describe some new understanding of interaction of parameters within the limitations of the model. A very successful example of this in the model is the analysis of the higher contribution of the Inoculated and Inoculated Infected states to dynamics and the introduction of another parameter, q, which limits the interaction of the Inoculated with other individuals. So, it explores the impact of the news states in the model and then introduces a specific way to mitigate their dangerous impact, which has important policy implications (granted that the underlying assumptions hold up). However, some aspects of the model cannot be applied directly to policy. Specifically, in several places of the manuscript the authors talk about the need for the determination of “optimal vaccination strategy”. While the model does explore vaccination rate as a parameter, I do not believe that their model is applicable to determination of a vaccine strategy at all. The reason for that is because a vaccine strategy considers some other parameters that are absent in the model. Specifically, an optimal vaccination strategy would consider, and probably prioritize, the short/medium term mortality rate and the hospital burden, something that is absent here.

**We agree, and have modified the text accordingly, explicitly indicating that the model presented does not contain enough detail to define an optimal vaccination strategy. In many places in the revised version, we still refer to the “MCI”, which is the vaccination rate yielding the minimum cumulative number of infections for the given parameter set. We emphasize in the revised version that the MCI does not necessarily correspond to an “optimal” strategy given the many additional factors to consider.**

 The design of the model in the confines of the classical SIR structure (where the pandemic geos through the population without any negative feedback loops leading to waves) also makes it unrealistic in providing an optimization of policy.

**We acknowledge this limitation and have briefly, but explicitly stated in the text that the reviewer’s work treats the impact of modeling cyclical transmission.**

 In sum, the conceptual description of the model, and the subsequent discussion, should avoid references to quantitative optimization of potential policy decisions that are likely to be influenced by parameters and outcomes that are not inherent in the model.

**We have generally avoided language referring to optimization in the revised version.**

 Minor issues:

 Equation of V’ has a missing parenthesis.

**We apologize for the oversight and thank the reviewer for bringing this to our attention.**

# Peer review: Reviewer response for version 1

**DOI:** 10.5256/f1000research.55607.r89322

**Published:** 2021-07-16

**Authors:** Sebastian Maximilian Schloer

**Affiliations:** 1 Center for Molecular Biology of Inflammation, Institute of Medical Biochemistry, University of Muenster, Muenster, Germany

In the manuscript entitled “Substantial impact of post-vaccination contacts on cumulative infections during viral epidemics”, the authors refer to the interaction between vaccination rate and limiting post-vaccination contacts for the course of an epidemic/pandemic.

 Naturally, this is a very relevant issue that deserves the careful attention of researchers working in this field. By modeling the epidemic/pandemic spreading of zoonotic diseases considering the vaccination rate may provide new insights for the future management of COVID-19.

 While study design and work are technically sound correct, the presentation of the results could be improved by adding figure legends (figure 2 C/D/E). To increase the readability of the data, it might be helpful to use a similar scale in figure 2 AB for easier comparison between the two different scenarios. The authors also assumed a very high vaccination rate for the calculation of epidemic/pandemic spreading, a lower vaccination rate (k _V_=0.005 like observed in the USA) would be better at covering a realistic influence of vaccination and post-vaccination contacts for the course of the epidemic. Thus, the author should include data obtained with a lower daily vaccination rate to verify the relevance of the model and results for the management of future epidemics/pandemics.

 The authors should also consider the pulsatile spreading and vaccination (that is normally, and currently observed in the SARS-CoV-2 pandemic) for zoonotic diseases and their management.

 In sum, this is a relevant and timely topic. The manuscript is well-written and the comprehensive overview of vaccination rate and limiting post-vaccination contacts for the management of the ongoing COVID-19 pandemic is clearly presented. I hope that the authors can expand the section by adding the required changes and considerations.

Is the work clearly and accurately presented and does it cite the current literature?

Yes

If applicable, is the statistical analysis and its interpretation appropriate?

Partly

Are all the source data underlying the results available to ensure full reproducibility?

Yes

Is the study design appropriate and is the work technically sound?

Yes

Are the conclusions drawn adequately supported by the results?

Yes

Are sufficient details of methods and analysis provided to allow replication by others?

Partly

Reviewer Expertise:

virology, pharmacology, zoonotic diseases

I confirm that I have read this submission and believe that I have an appropriate level of expertise to confirm that it is of an acceptable scientific standard, however I have significant reservations, as outlined above.

# Response

**Published:** 2021-08-16

**Authors:** Eugene Koonin

**Affiliations:** National Center for Biotechnology Information (NCBI), National Institutes of Health (NIH), USA

**We thank the reviewer for their kind and constructive remarks.**

 While study design and work are technically sound correct, the presentation of the results could be improved by adding figure legends (figure 2 C/D/E).

**Legends have been added.**

 To increase the readability of the data, it might be helpful to use a similar scale in figure 2 AB for easier comparison between the two different scenarios.

**We appreciate the reviewer’s suggestion; however, we found if we set the color scale to run from 0 to 1 for both panels, the variation in Figure 2B is hard to see with the same colormap. We have elected to keep separate scales but have modified the Figure description to emphasize that the color scales are different.**

 The authors also assumed a very high vaccination rate for the calculation of epidemic/pandemic spreading, a lower vaccination rate (kV=0.005 like observed in the USA) would be better at covering a realistic influence of vaccination and post-vaccination contacts for the course of the epidemic. Thus, the author should include data obtained with a lower daily vaccination rate to verify the relevance of the model and results for the management of future epidemics/pandemics.

**We apologize for any confusion. Figures 2A/B/C display results for a range of vaccination rates from 1e-5 to 3e-2. Figure 2D displays two fixed rates 0.01 and 0.03 in addition to the vaccination rate which yields the fewest cumulative number of infections which in some parameter regimes is approximately the rate suggested by the reviewer. Figure 3 displays both a fixed rate of 0.03 and the vaccination rate which yields the smallest cumulative number of infections. Regarding realism for the choice of fixed rates, we acknowledge and state in the text that 0.03 represents a high rate of vaccination; however, we also do not attempt (and have modified the language in the revised version to this effect) to make empirical projections which could be explicitly interpreted in the context of any specific epidemic. We do not model age structure, for example, which significantly affects both the natural history of SARS-CoV-2 as well as the vaccine rollout (administered to older individuals first). Our goal in selecting the range of rates displayed was to demonstrate that the dependence on the cumulative number of infections on vaccine distribution rate is nonmonotonic within a nontrivial parameter regime (the majority of the population is vaccinated over a period of months to a year).**

 The authors should also consider the pulsatile spreading and vaccination (that is normally, and currently observed in the SARS-CoV-2 pandemic) for zoonotic diseases and their management.

**We appreciate the reviewer’s input. Incorporating pulsatile or periodic forcing into the model would require significant changes to be made which we feel are outside the scope of the current work. We do, however, cite Rella *et al. *(2021)** **and have modified the text to indicate that cyclical models are addressed in this reference.**

 In sum, this is a relevant and timely topic. The manuscript is well-written and the comprehensive overview of vaccination rate and limiting post-vaccination contacts for the management of the ongoing COVID-19 pandemic is clearly presented. I hope that the authors can expand the section by adding the required changes and considerations.

**We thank the reviewer for their interest and hope they find the revised version satisfactory.**
